# Mechanisms of resistance to CAR-T cell therapy in multiple myeloma: latest updates from the 2024 ASH annual meeting

**DOI:** 10.1186/s40164-025-00643-6

**Published:** 2025-03-26

**Authors:** Yuhan Hu, Jiangxue Hou, Zhongxing Jiang, Quande Lin

**Affiliations:** 1 The First Affiliated Hospital of Zhengzhou University, Zhengzhou, 450000 China; 2 The Affiliated Cancer Hospital of Zhengzhou University & Henan Cancer Hospital, Zhengzhou, 450008 China

**Keywords:** CAR-T, Multiple myeloma, Antigen escape, Immune suppression, CAR-T cell exhaustion, Mechanisms

## Abstract

Chimeric Antigen Receptor T-Cell (CAR-T) therapy demonstrates significant potential in the treatment of multiple myeloma (MM). However, resistance to CAR-T therapy remains a critical challenge. Investigating the mechanisms underlying CAR-T resistance, including antigen escape, immunosuppression, and CAR-T cell exhaustion, is essential for enhancing the long-term efficacy of this therapeutic approach. This study provides an in-depth review of the latest findings presented at the 66th ASH Annual Meeting.

To the editor:

CAR-T therapy targeting BCMA has revolutionized the treatment landscape for relapsed/refractory MM patients, achieving remarkable initial responses. However, mechanisms of relapse/resistance, such as antigen escape, CAR-T cell exhaustion, and immunosuppressive tumor microenvironment, continue to impede its long-term efficacy. In this review, we present a comprehensive summary of the latest findings from the 66th ASH Annual Meeting.

## Antigen escape

1. The study by Francesco Maura et al. investigates genomic profiling in patients who relapse following CAR-T therapy [1]. Key resistance mechanisms include BCMA (TNFRSF17) loss, alterations in NF-κB pathway genes (e.g., CYLD, TRAF3), and markers of genomic instability such as TP53 and CDKN2C mutations. These genetic alterations, often pre-existing, contribute to primary refractoriness. Notably, biallelic BCMA loss is only tolerated alongside NF-κB pathway disruptions, collectively driving drug resistance. The study further demonstrates that genomic analysis is a more effective predictor of CAR-T therapy refractoriness compared to traditional clinical risk factors, including extramedullary disease and the MyCARe score.
2. The study by David Jung et al. demonstrates that TRAF3 deletion activates the non-canonical NF-κB signaling pathway, leading to upregulation of anti-apoptotic proteins such as MCL-1 and BCL-2, thereby enhancing tumor survival and resistance to anti-BCMA CAR-T therapy [2]. Interestingly, TRAF3 knockout (KO) does not significantly alter BCMA surface expression but reduces myeloma cell sensitivity to anti-BCMA CAR-T therapy in vitro. Genomic and functional analyses reveal that NF-κB activation—via TRAF3 deletion or mutations in related genes like CYLD and MAP3K14, drives acquired resistance to BCMA-targeted immunotherapies. Clinical data further confirm this mechanism, with frequent NF-κB mutations observed in resistant cases.
3. Another study explored the molecular mechanisms of CAR-T resistance using genomic and epigenetic analyses, identifying a resistant tumor plasma cell subpopulation, Cluster 5 (C5), which survives treatment and contributes to relapse [3]. Gene expression analysis of C5 cells revealed differential expression of genes such as MS4A1, HLA-DPB1, and CD74, are associated with antigen processing and the CD74 signaling pathway. Cell-cell communication analysis showed stronger interactions between tumor plasma cells and T lymphocytes, particularly through MHC-II, MIF, CD74, and LT signaling pathways. Co-culture experiments confirmed increased CD74 expression on MM cells after CAR-T exposure, suggesting its role in resistance.

## Immunosuppressive tumor environments

1. Studies have shown that S100A8/A9 (calprotectin) is highly expressed in myeloid-derived suppressor cells (MDSCs) from the bone marrow of MM patients, highlighting its role in immune suppression [4]. In vitro, exogenous S100A8/A9 dose-dependently inhibited the cytotoxic activity of anti-BCMA CAR-T cells against MM1S cells. This inhibition was linked to reduced CAR-T cell activation, as evidenced by decreased CD69 expression, and diminished production of cytokines, such as IFN-γ, IL-2, TNF-α, and GM-CSF. Additionally, S100A8/A9 exposure upregulated inhibitory checkpoint markers TIGIT and TIM3 on CAR-T cells, promoting their differentiation into IL-10-secreting regulatory T cells (Tregs), which further impaired CAR-T efficacy in co-culture experiments.
2. The role of Platelet-Activating Factor (PAF) in MM relapse following CAR-T therapy was discussed at this meeting. Among CAR-T-treated patients, 63% experienced relapse [5]. Relapsed patients showed decreased phospholipids in the lysophosphatidylcholine (LPC) and PAF metabolic pathways, elevated plasma PAF levels, and increased PAF receptor (PAFR) protein expression in the bone marrow. In vitro, PAF treatment significantly increased MM cell proliferation without affecting CAR-T cell proliferation or cytotoxicity. These findings indicate that the upregulation of the PAF pathway after CAR-T therapy enhances MM cell proliferation and contributes to relapse, while not directly impairing CAR-T cell function.
3. The bone marrow-derived macrophages (BMDMs) polarized by MM cells significantly impaired CAR-T cell cytotoxicity, while naive, unstimulated BMDMs had no impact [6]. This highlights the role of tumor-associated macrophages (TAMs) in immune suppression and CAR-T therapy resistance. The interaction between MM cells and macrophages, particularly via the complement-related C1q/C1qbp pathway, induced an immunosuppressive response and increased pro-inflammatory chemokine production, further driving resistance. Although CAR-T treatment reduced C1q-expressing macrophages, their persistence or resurgence in the bone marrow may contribute to relapse. Notably, C1q + macrophages exhibited upregulation of markers such as TYROBP and FCER1G, which are associated with macrophage polarization and infiltration.
4. It was revealed that the bone marrow immune microenvironment is critical to treatment failure in CAR-T therapy [7]. A major factor contributing to this failure is the expansion of MDSCs post-infusion, which correlates with adverse prognosis. The study underscores the complexity of immune dynamics, highlighting the regulatory roles of immune checkpoints (PD-1, TIGIT, TIM-3) and regulatory Tregs in CAR-T cell responses. Additionally, altered expression of molecules such as CD74, S100A8/A9, and macrophage migration inhibitory factor (MIF) which mediate interactions between myeloma cells and the immune system further contributes to resistance.

## CAR-T cell exhaustion

T-cell exhaustion, marked by reduced cytotoxicity and functional decline in CD8 + T cells, is a major factor in relapse among RRMM patients treated with CAR-T therapy [8]. Overexpression of immune checkpoint molecules, particularly TIGIT, on both CAR-T and tumor-infiltrating T cells, was linked to functional impairment and disease progression. Additionally, gene expression profiling in early-relapse patients revealed disrupted pathways related to T-cell activation, neutrophil regulation, and leukocyte proliferation, indicating that exhaustion-prone T-cell phenotypes prior to CAR-T infusion may predispose patients to relapse.

## Conclusion

Resistance to CAR-T cell therapy in multiple myeloma is driven by tumor-intrinsic factors, immune checkpoint dysregulation, and an immunosuppressive microenvironment (Table 1) [9–12]. Strategies aimed at overcoming T-cell exhaustion, enhancing CAR-T persistence, and targeting immune-suppressive pathways offer novel insights into potential therapeutic interventions.

**Table 1:** Mechanisms of resistance to CAR-T therapy in multiple myeloma

Study Mechanism	Target	Key Findings	Reference
Antigen Escape	Genomic Instability	BCMA loss and NF-κB(CYLD, TRAF3) disruptions contribute to drug resistance.	[1]
	NF-κB Activation	TRAF3 deletion induces non-canonical NF-κB pathway, upregulating anti-apoptotic proteins (MCL-1, BCL-2) and conferring resistance to anti-BCMA CAR-T.	[2]
	CD74 Pathway	Tumor plasma cells cluster with upregulated CD74 expression contributing to CAR-T resistance. MIF/CD74 signaling pathway inhibits CAR-T efficacy.	[3]
Immunosuppressive Tumor Environments	S100A8/A9	S100A8/A9 in MDSCs reduces CAR-T activation and cytokine production, promotes Treg differentiation, and suppresses CAR-T cytotoxicity.	[4]
	PAF Pathway	Elevated PAF levels and PAF receptor expression contribute to MM cell proliferation after CAR-T therapy, promoting relapse without affecting CAR-T cell function.	[5]
	Macrophage Polarization	TAMs, particularly C1q-expressing macrophages, inhibit CAR-T activity.	[6]
	the expansion of MDSCs	The expansion of MDSCs is a critical factor influencing treatment efficacy and relapse in CAR T-cell therapy.	[7]
CAR-T Cell Exhaustion	T-cell Exhaustion	T-cell exhaustion, characterized by increased TIGIT expression on CD8 + T cells, impairs CAR-T cell function.	[8]

## Data Availability

No datasets were generated or analysed during the current study.

## Abbreviations

CAR-T: Chimeric Antigen Receptor T-Cell
MM: Multiple myeloma
KO: Knockout
MDSCs: Myeloid-derived suppressor cells
Tregs: Regulatory T cells
PAF: Platelet-Activating Factor
LPC: Lysophosphatidylcholine
PAFR: PAF receptor
MIF: Migration inhibitory factor
